# Opening of New Jewish Wards at the London Hospital

**Published:** 1904-11-19

**Authors:** 


### OPENING OF NEW JEWISH WARDS AT THE LONDON HOSPITAL.

The new wards for Jewish patients, provided by the generosity of the late Mr. E. L. Raphael, were opened on Monday by Mr. Leopold de Rothschild, in the presence of a large and distinguished number of visitors. A consecration service, partly in Hebrew and partly in English, having been conducted by the Rev. S. Singer and the Rev. S. Levy, M.A., Mr. Leopold de Rothschild, in the absence of Lord Rothschild, declared the wards open. The Hon. Sydney Holland, in moving a vote of thanks, expressed his regret that Lord Rothschild, as the head of a family whose generosity, never appealed to in vain, was not confined to helping those of their own faith, was unavoidably prevented from performing the ceremony, and also that Mr. Adler, the Chief Rabbi, was not able to be present. As showing the need for adequate accommodation for the Jewish community, especially in the East End, Mr. Holland mentioned that during 1903, 1,540 in-patients and 45,000 out-patients, in addition to 1,800 maternity cases, belonged to the Jewish faith. Whereas these in-patients had been scattered in the various wards, the number of beds in the old wards being limited to 27, they would now be gathered together in four well-equipped wards, two for men and two for women, this improvement having been effected without curtailing any of the hospital's activity. Indeed his great regret was that the completion of the ward given by Mr. Raphael in memory of his wife (for which he had subscribed £20,000) was delayed through the anxiety of both the hospital authorities and the donor to avoid turning patients away. He considered these wards the very best in the hospital, and he wished to draw special attention to the way in which the difficulty of ventilation had been surmounted by the architect, Mr. Rowland Plumbe.

The new wards are on the top floor of the west wing of the hospital, and provide accommodation for 27 men and 27 women—in four wards, each about 72 feet long, 21 feet wide, and 12 feet high. There is a large kitchen, centrally situated between the wards, and used exclusively for them. Each ward has a sister's room, scullery, bath-room, etc. A wide balcony runs round the south end, and there is a smaller and separate balcony at the side of each ward, approached by means of glazed folding doors. The floors of the wards are finished with cement and covered with linoleum, and are warmed with "Teale's" stoves.

The kitchen, scullery, corridors, lavatories, etc., are paved with mosaic, and the walls are tiled.

The work has been carried out for the Governors by Messrs. Perry and Company, of Bow, under the direction of Messrs. Rowland Plumbe and Harvey, of 13 Fitzroy Square, the architects for the rebuilding of the hospital.

